# Characteristics of Adolescents With Elevated Suicide Risk Presenting to the ED With Physical Health Complaints

**DOI:** 10.1111/acem.70307

**Published:** 2026-04-28

**Authors:** Polina Krass, Renata A. Sanders, Abigail M. Ross, Evan M. Dalton, Priyanka Joshi, Joel A. Fein, Stephanie K. Doupnik

**Affiliations:** ^1^ Division of Emergency Medicine Children's Hospital of Philadelphia Philadelphia Pennsylvania USA; ^2^ PolicyLab Children's Hospital of Philadelphia Philadelphia Pennsylvania USA; ^3^ Center for Violence Prevention Children's Hospital of Philadelphia Philadelphia Pennsylvania USA; ^4^ Leonard Davis Institute for Health Economics University of Pennsylvania Philadelphia Pennsylvania USA; ^5^ Department of Pediatrics University of Pennsylvania Philadelphia Pennsylvania USA; ^6^ Division of Adolescent Medicine Children's Hospital of Philadelphia Philadelphia Pennsylvania USA; ^7^ School of Social Policy and Practice University of Pennsylvania Philadelphia Pennsylvania USA; ^8^ Department of Psychiatry, Perelman School of Medicine University of Pennsylvania Philadelphia Pennsylvania USA; ^9^ Department of Pediatrics, Baylor College of Medicine Texas Children's Hospital Houston Texas USA; ^10^ Departments of Pediatrics and Health Policy Vanderbilt University Medical Center Nashville Tennessee USA

## 1 Characteristics of Adolescents with Elevated Suicide Risk Presenting to the ED with Physical Health Complaints

Suicide is the second leading cause of death in United States (US) adolescents [1]; 10% of adolescents attempt suicide annually and more than 20% of adolescents seriously consider suicide [2]. Emergency Departments (EDs) are a crucial location for screening and identification of adolescent suicide risk, as patients presenting to EDs are less likely to have other sources of care and more likely to have health‐related social needs that may put them at risk for mental health emergencies [3, 4]. US Joint Commission guidelines recommend ED suicide risk screening only for patients being evaluated for a mental health condition [5], and many EDs do not routinely screen for suicide risk in adolescents presenting with physical health complaints [6, 7, 8]. We aimed to identify the characteristics and presenting chief complaints of adolescents who presented to the ED with a physical health chief complaint and were found to have acutely elevated risk of suicide on routine universal ED suicide screening.

We identified all ED visits for youth aged 12–21 presenting to a single‐center, urban pediatric ED between June 2013 and May 2024 who completed the Behavioral Health Screen‐ED [9], a psychometrically validated, self‐administered digital survey used for routine universal suicide and behavioral risk screening of all patients aged ≥ 12 in this ED. Patients considered too ill by nursing staff, those with severe intellectual disability that precluded survey completion, and those who did not speak English (or Spanish from 2022 onwards) were excluded from screening. The BHS‐ED includes 3 two‐part questions about suicide risk. If a patient endorses lifetime suicidal ideation, plan, or attempt, they are then asked a follow‐up question about risk within the past week:

1. Have you ever thought about killing yourself? (In the past week, including today, have you thought about killing yourself?)
2. Did you ever make a plan to kill yourself? (In the past week, including today, did you have a plan to kill yourself?)
3. Have you ever tried to kill yourself? (In the past week, including today, have you tried to kill yourself?)

Our primary outcome, past week suicidal thoughts or behaviors (past week STB), was defined as any endorsement of suicidal thoughts, plans, and/or attempts within the past week.

We included all ED encounters. For patients who had multiple visits during the study period, each encounter with a completed BHS‐ED was included. We extracted self‐reported gender, race and ethnicity data from the BHS‐ED, and we extracted age, insurance coverage type, chief complaint, triage level, and disposition from the electronic health record.

We classified presenting complaints into mental health‐related and physical health‐related. We defined mental health‐related chief complaints using internally validated search term schema, which included the terms “Psychiatric Emergency” or “Suicide”, any specific DSM‐10 diagnoses (such as Anxiety, Depression, Suicide, Anorexia), and any mental health‐related symptoms (Supporting Information).

Physical health chief complaints were grouped using a modified version of the Pediatric Emergency Reason for Visit Clusters (Supporting Information) [10]. We excluded patients for whom the listed chief complaint was “Other” or “Evaluation and Treatment”, as it was unclear if these patients were presenting for primarily physical or mental health reasons.

Among patients with a physical health chief complaint, we used multivariable logistic regression clustered by patient to evaluate the odds of past week STB by patient and visit characteristics. In evaluating chief complaint categories, we used “Fever” as the referent group as fever is common and is unlikely to be influenced by past week STB. For all other variables, we used the category with the lowest odds of past week STB as the reference group, allowing relative differences between categories to be more clearly interpreted.

This study followed the STROBE guidelines for cross‐sectional studies. As a secondary analysis of deidentified data, this study was determined not to be Human Subjects Research by the Institutional Review Board at our institution. We performed all data analysis using Stata 18.5 (College Station, TX). Our statistical code is publicly available (Supporting Information) and deidentified data may be shared upon request.

There were 65,705 ED visits for patients aged 12–21 with completed suicide risk screening out of 165,696 total age‐ and language‐eligible ED visits between 2013 and 2024 (40%, Table S2). Screened patients had higher odds of identifying as female or Hispanic/Latino, being aged 12–13, and presenting for a mental health‐related chief complaint.

Among visits with completed suicide risk screening, 49,621 (75.5%) ED visits were for a physical health complaint, representing 29,921 unique patients. There was a median of two ED visits over the study timeframe per patient (interquartile range, 1–3), and 83.3% of visits resulted in discharge. Past week STB was identified in 1179 (2.4%) visits with a physical health complaint; past‐week suicide attempt was the highest risk category disclosed in 489 (41.5%) visits, with the remainder disclosing plan (*n* = 253, 21.5%) or ideation (*n* = 437, 37.1%) (Table 1). Among patients with past week STB, 64.3% were discharged from the ED; the remainder were admitted or transferred.

**TABLE 1:** ED visits for patients aged 12–21 with past‐week suicidal thoughts or behaviors detected on routine screening at a Children's Hospital ED, 2013–2024^a^.

	ED visits with a physical health chief complaint		Adjusted odds ratio^b^
	All visits with behavioral health screening	Past week suicidal thoughts or behaviors identified	
*n*	49,621	1179	
Most frequent chief complaints among youth with suicide risk, *n* (%)			
Poisoning	394 (0.8%)	146 (12.4%)	**25.12 (14.99–42.11)**
Abdominal Pain	8273 (16.7%)	144 (12.2%)	1.37 (0.84–2.24)
Injury/Trauma	6015 (12.1%)	142 (12.0%)	**2.14 (1.29–3.57)**
Sexually transmitted infection	1140 (2.3%)	129 (10.9%)	**8.80 (5.33–14.52)**
Headache	4635 (9.3%)	78 (6.6%)	1.30 (0.80–2.12)
Respiratory distress	4066 (8.2%)	73 (6.2%)	1.17 (0.71–1.93)
Chest pain	4220 (8.5%)	61 (5.2%)	1.07 (0.64–1.82)
GI symptom	3335 (6.7%)	52 (4.4%)	1.20 (0.70–2.05)
Fainting/Syncope	1880 (3.8%)	38 (3.2%)	1.37 (0.77–2.43)
ENT Symptom	2590 (5.2%)	37 (3.1%)	1.33 (0.80–2.19)
Child abuse/Sexual assault	273 (0.6%)	33 (2.8%)	**4.62 (2.49–8.57)**
Urinary symptom	1284 (2.6%)	31 (2.6%)	**1.99 (1.09–3.65)**
Dermatologic	1912 (3.9%)	27 (2.3%)	1.15 (0.63–2.12)
Diabetes	513 (1.0%)	23 (2.0%)	**2.01 (1.07–3.79)**
Feeding/Eating issues	951 (1.9%)	21 (1.8%)	2.04 (1.06–3.93)
Fever	1507 (3.0%)	19 (1.6%)	Reference
Patient characteristics, *n* (%)			
Age			
12–13	4603 (9.3%)	178 (15.1%)	**1.82 (1.30–2.55)**
14–15	17,915 (36.1%)	480 (40.7%)	**1.42 (1.05–1.92)**
16–17	20,136 (40.6%)	405 (34.4%)	**1.16 (0.87–1.53)**
18–21	6967 (14.0%)	116 (9.8%)	Reference
Gender			
Female	33,389 (67.3%)	914 (77.5%)	**2.34 (1.93–2.85)**
Male	15,085 (30.4%)	197 (16.7%)	Reference
Transgender	708 (1.4%)	59 (5.0%)	**6.16 (3.84–9.90)**
Not reported^c^	439 (0.9%)	9 (0.8%)	**6.87 (3.15–14.98)**
Race			
Black	24,959 (50.3%)	604 (51.2%)	**1.51 (1.19–1.91)**
White	15,334 (30.9%)	253 (21.5%)	Reference
More than one race	5187 (10.5%)	201 (17.1%)	**2.04 (1.53–2.71)**
Asian	1411 (2.8%)	28 (2.4%)	1.24 (0.74–1.95)
Other specified^d^	486 (1.0%)	24 (2.0%)	**3.16 (1.83–5.45)**
Not reported^c^	2224 (4.5%)	69 (5.9%)	**1.81 (1.21–2.69)**
Ethnicity			
Not Hispanic or Latino	41,411 (83.5%)	894 (75.8%)	Reference
Hispanic or Latino	5605 (11.3%)	193 (16.3%)	1.23 (0.97–1.63)
Not reported^c^	2605 (5.2%)	92 (7.8%)	**1.46 (1.10–1.92)**
ED Visit Characteristics, *n* (%)			
Triage level			
1 (Critical)	45 (0.1%)	6 (0.5%)	**9.85 (3.78–25.70)**
2 (Acute)	7762 (15.6%)	460 (39.0%)	**3.39 (2.66–4.32)**
3 (Urgent)	29,217 (58.9%)	495 (42.0%)	1.14 (0.91–1.43)
4 (Urgent)	12,144 (24.5%)	207 (17.6%)	Reference
5 (Non‐urgent)	451 (0.9%)	11 (0.9%)	1.65 (0.74–3.67)
Insurance, *n* (%)			
Medicaid	26,254 (52.9%)	713 (60.5%)	1.24 (1.03–1.47)
Private	21,732 (43.8%)	416 (35.3%)	Reference
Self‐Pay or not listed	1635 (3.3%)	50 (4.2%)	—

^a^ Row percentages may not sum to 100% due to rounding.

^b^ Multivariable odds of reporting past‐week suicide ideation, plan or attempt, followed by confidence interval, adjusted by all other listed characteristics and clustered by patient. Significant findings in bold.

^c^ Not reported includes “I don't know the answer”, “I don't want to answer,” “not sure” or missing.

^d^ Other Specified category includes American Indian, Alaska Native, Native Hawaiian or Other Pacific Islander.

By proportion, the five most common chief complaints among ED encounters for patients found to have past week STB were Poisoning (12.4%, *n* = 146); Abdominal Pain (12.2%, *n* = 144); Injury/Trauma (12.0%, *n* = 142); STI (10.9%, *n* = 129); and Headache (6.6%, *n* = 78) (Table 1 and Supporting Information). Compared to a chief complaint of Fever, patients had significantly higher odds of presenting with past week STB with a chief complaint of Poisoning (aOR 25.12, CI 14.99–42.11); Child Abuse/Sexual Assault (aOR 4.62, CI 2.49–8.57); STI (aOR 8.80, CI 5.33–14.52); Foreign Body (aOR 7.49, CI 3.54–15.84); Diabetes (aOR 2.01, CI 1.07–3.79); Urinary Symptom (aOR 1.99, CI 1.09–3.65); or Injury/Trauma (aOR 2.14, CI 1.29–3.57).

Adjusting for other patient and ED visit characteristics, patients with past week STB had higher odds of identifying as Transgender (aOR 6.44, CI 4.12–10.07) or Female (aOR 2.40, CI 1.99–2.91), versus Male; identifying as Black (aOR 1.51, CI 1.21–1.89), More than one race (aOR 2.03, CI 1.55–2.67), or Other specified (comprised of American Indian, Alaska Native, Native Hawaiian, or Pacific Islander, aOR 3.20, CI 1.89–5.43), versus White; and having Medicaid insurance, versus private insurance (aOR 1.22, CI 1.03–1.44). Compared to 18–21 year olds, younger patients had higher odds of reporting past week STB (Ages 12–13: aOR 2.57, 95% CI 1.83–3.59; Ages 14–15: aOR 1.86, 95% CI 1.37–2.51; Ages 16–17 years: aOR 1.41, 95% CI 1.06–1.87).

In this study of nearly 50,000 pediatric ED visits over an 11‐year period, 2.4% of ED encounters for adolescents presenting to the ED with a physical health chief complaint were found to have current past week STB, across chief complaints and patient characteristics. Prior work suggests that one in five adolescents presenting to the ED report lifetime or current suicide risk, regardless of chief complaint, although the published range is wide (4%–29%) [6, 11]. In this study of youth presenting with physical health complaints only, and using a narrowed definition of suicide risk (past‐week rather than lifetime), we identified past‐week STB in more than 1 in 50 adolescents and a past‐week suicide attempt in 1 in 100. These findings support universal ED screening as a strategy to improve detection of adolescent suicide risk. We contribute additional detail about the types of patients who may be detected using a universal screening approach. More than a third of patients who reported past week STB in our sample presented with a non‐specific chief complaint: abdominal pain, chest pain, respiratory distress, or a GI symptom. Additionally, patients with occult suicide risk were more likely to identify as Transgender or Female, non‐White, and to have Medicaid insurance. This highlights the value of universal suicide screening, as targeted approaches may miss a significant proportion of at‐risk youth and may underrecognize youth from minoritized or economically disadvantaged backgrounds.

We identified several chief complaints that were associated with past week STB. Presenting with Child Abuse/Sexual Assault was associated with a nearly 5‐fold increased odds of past week STB, consistent with the well‐established risk of suicide in individuals with experiences of abuse [12]. We found nearly 9‐fold increased odds of past week STB in patients presenting with a STI concern, similar to a study examining presenting complaints of patients presenting to primary care with a history of suicide attempt [13]. A chief complaint of Poisoning was associated with more than 25‐fold odds of past week STB, aligning with a recent study demonstrating that pediatric ED patients presenting with unintentional poisoning had significantly greater odds of elevated suicide risk screening [14]. These findings indicate that patients presenting with abuse, sexual health concerns, ingestion, intoxication and/or overdose warrant additional consideration, even if suicidality is not initially disclosed.

This study has limitations. As a single‐center study in a freestanding pediatric ED, the findings may not be generalizable to other EDs. The suicide screening questions asked in this study (BHS‐ED) may differ in accuracy or sensitivity from other evidence‐based screening tools. At our institution, the positive percent agreement between the BHS‐ED and the Ask Suicide Questions (ASQ) for patients who complete both is high (96.9%). Similar to other studies of self‐administered ED screens [15], our analysis does not include visits for patients with intellectual disability or medical acuity that precluded self‐administered screening, those who declined screening, those for whom the BHS‐ED was not available in their preferred language, or those discharged before completing the screen. Patients who did not complete screening may differ systematically from studied respondents (Table S2). Finally, patients with physical health chief complaints may have had intentional self‐harm or suicide attempt identified as part of routine care. Even in these situations, universal suicide screening may help to mobilize resources such as safety observation earlier in the ED visit and prompt earlier evaluation for co‐occurring risks.

In conclusion, universal suicide risk screening identified that 2.4% of adolescents who presented to the ED for a physical health complaint had suicidal ideation, plan, or attempt in the past week. These findings support universal ED screening as a key strategy to detect adolescent suicide risk. However, prioritizing enhanced screening for patients with chief complaints associated with elevated suicide risk may provide a pragmatic, complementary approach to improve suicide risk detection and enable potentially life‐saving intervention in the ED.

## Author Contributions

P.K. designed the study, collected and analyzed the data, and drafted the manuscript. R.A.S, A.M.R, E.M.D, and P.J. contributed to study design, interpretation of the data, and critically reviewed the manuscript. J.A.F. supervised acquisition of the data, contributed to analysis and interpretation of the data, and critically reviewed and revised the manuscript. S.K.D. contributed to study design, assisted with data analysis and interpretation, and critically reviewed and revised the manuscript. All authors approved the final version of the manuscript.

## Funding

The authors have nothing to report.

## Conflicts of Interest

The authors declare no conflicts of interest.

## Supporting information


**Table S1:** Study modifications to the Pediatric Reason for Visit Clusters (PERC).
**Table S2:** Comparison of the Analytic Sample to the Overall Emergency Department Population (2013–2024).
**Table S3:** Frequency of Physical Health Chief Complaints Among Youth Presenting to the ED with Past‐Week Suicidal Thoughts or Behaviors Detected on Routine Screening, *n* (%).

## Data Availability

The data that support the findings of this study are available from the corresponding author upon reasonable request, and in accordance with Institutional Data Use Agreement guidelines.
