# Supplementary material for: Characteristics of Adolescents With Elevated Suicide Risk Presenting to the ED With Physical Health Complaints
Source: Acad Emerg Med. 2026 Apr 28;33:e70307. doi: 10.1111/acem.70307 (PMC13125736; doi:10.1111/acem.70307)
Supplement: Supplementary file 1 — Table S1: Study modifications to the Pediatric Reason for Visit Clusters (PERC). Table S2: Comparison of the Analytic Sample to the Overall Emergency Department Population (2013–2024). Table S3: Frequency of Physical Health Chief Complaints Among Youth Presenting to the ED with Past‐Week Suicidal Thoughts or Behaviors Detected on Routine Screening, n (%). [file ACEM-33-0-s001.docx]

**Supplemental Materials**

Our full statistical code, including our chief complaint categorization algorithm, is available at github.com/krassp.

**Supplemental Table 1.** Study modifications to the Pediatric Reason for Visit Clusters (PERC)^a^

| **Original PERC Cluster** | **Modified PERC Cluster** |
| --- | --- |
| Abdominal pain | No modification |
| Abuse/assault | Renamed "Child abuse/Sexual assault" |
| Allergic reaction | No modification |
| Altered mental status | No modification |
| Asthma/wheezing | Grouped into "Respiratory distress" |
| Bites/stings | No modification |
| Burn | Grouped into "Dermatologic" |
| Cardiac complaint | No modification |
| Chest pain | No modification |
| Chronic disease | Separated into “Diabetes”, “Sickle Cell Anemia” and “Oncolgic” |
| Congestion/URI | Grouped into "ENT Symptom" |
| Constipation | Grouped into "GI Symptom" |
| Cough | Grouped into "Respiratory Distress" |
| Croup | No modification |
| Crying/colic | No modification |
| Dental complaint | No modification |
| Device complication | No modification |
| Diarrhea | Grouped into "GI Symptom" |
| Ear complaints | Grouped into "ENT Symptom" |
| Epistaxis | Grouped into "ENT Symptom" |
| Extremity injury or pain | Extremity injury grouped with "Injury/Trauma"; "Limp/Gait issue" and "Extremity pain" categorized separately |
| Eye complaints | No modification |
| Fainting/syncope | No modification |
| FB (ENT/GI) | Grouped into "Foreign body" |
| FB (skin) | Grouped into "Foreign body" |
| Fever | No modification |
| Fever in a neonate | No modification |
| Follow-up/recheck | No modification |
| General/nonspecific symptoms | No modification |
| GI bleeding | Grouped into "GI Symptom" |
| Gynecologic | No modification |
| Head or neck injury | Grouped into "Injury/Trauma" |
| Headache | No modification |
| Laceration | Grouped into "Dermatologic" |
| Lump/mass | Grouped into "Dermatologic" |
| Male genital | No modification |
| Motor vehicle collision | Grouped into "Injury/Trauma" |
| Neck pain | No modification |
| Other | No modification |
| Other neurologic | No modification |
| Poisoning | No modification |
| Poor feeding/mouth complaint | No modification |
| Pregnancy | No modification |
| Primary care | No modification |
| Psychiatric/behavioral | Categorized the following search terms (in any part of phrase) as psychiatric/behavioral:  Anxiety  Panic  Psych  Suicide  Depression  Depressed  Behavioral  Behavior  Aggression  Aggressive  Self-injury  Bulimia  Anorexia  BH referral |
| Rash | Grouped into "Dermatologic" |
| Respiratory (other) | No modification |
| Seizure | No modification |
| Sore throat | Grouped into “ENT symptom” |
| Trauma -- multiple, other, or unspecified | Grouped into "Injury/Trauma" |
| Urinary symptoms | No modification |
| Vomiting | Grouped into "GI Symptom" |
| No PERC equivalent | Jaundice |
| No PERC equivalent | Sexually Transmitted Infection |

1. Original PERC clusters developed in Gorelick MH, Alpern ER, Alessandrini EA. “A system for grouping presenting complaints: the pediatric emergency reason for visit clusters.” Acad Emerg Med 2005;12(8):723–31. Key modifications made for this analysis include: subcategorizing the “Chronic Disease” cluster into specific disease entities (eg Diabetes, Oncologic, Sickle Cell Anemia), creating a new category for Sexually Transmitted Infection (STI), rather than grouping this concern under gender-specific clusters (e.g., male or female genitourinary), and grouping specific symptoms into organ-based categories as aligned with our Electronic Medical Record documentation.

**Supplemental Table 2. Comparison of the Analytic Sample to the Overall Emergency Department Population (2013-2024)**

|  | ED encounters for age and language-eligible patients during the study period^a^ | ED encounters during which Behavioral Health Screening (BHS-ED) was completed during the study period | Odds of receiving screening^d^ |
| --- | --- | --- | --- |
| N | 165,696 | 66,705 |  |
| Race^b^ |  |  |  |
| Black/African American | 90,757 (55%) | 31,993 (48%) | - |
| White | 57,544 (35%) | 22,219 (33%) | - |
| Other | 13,363 (8%) | 636 (1%) | - |
| Asian | 4,032 (2%) | 2,137 (3%) | - |
| More than one^c^ |  | 7,047 (11%) | - |
| Not sure^c^ |  | 2,673 (4%) | - |
|  |  |  |  |
| Age |  |  |  |
| 12-13 | 13,944 (8%) | 7,483 (11%) | Reference |
| 14-15 | 65,532 (40%) | 24,173 (36%) | 0.50 (0.49-0.52_ |
| 16-17 | 62,174 (38%) | 26,231 (39%) | 0.63 (0.61-0.65) |
| 18-21 | 24,046 (15%) | 8,488 (13%) | 0.47 (0.45-0.49) |
| Ethnicity^b^ |  |  |  |
| Not Hispanic or Latino | 151,046 (91%) | 55,787 (84%) | Reference |
| Hispanic or Latino | 12,910 (8%) | 7,650 (11%) | 2.48 (2.39-2.58) |
| Unknown | 1,740 (1%) |  | - |
| Not sure^c^ |  | 3,268 (5%) | - |
| Sex/Gender^b^ |  |  |  |
| Female | 95,083 (57%) | 45,044 (68%) | Reference |
| Male | 70,598 (43%) | 20,034 (30%) | 0.44 (0.43-0.45) |
| Unknown | 15 (<1%) |  | - |
| Transgender^c^ |  | 1,403 (2%) | - |
| Not sure or don’t want to say^c^ |  | 223 (<1%) | - |
| Chief complaint |  |  |  |
| Psychiatric | 9,908 (6%) | 9,002 (14%) | 17.01 (15.86-18.21) |
| Non-Psychiatric | 155,788 (94%) | 57,472 (87%) | Reference |

1. BHS-ED has been offered standardly to all patients aged 12+ since October 2022. Between June 2013-April 2023, the BHS-ED was only offered to patients aged 14+ routinely. BHS-ED has been offered in Spanish since December 2022. Before then, it was only offered in English.
2. Data collected from the electronic medical record (EMR) for patients without a BHS-ED, and self-reported for patients with a BHS-ED.
3. Category only exists in the BHS-ED; no equivalent in the EMR.
4. Odds calculated using univariate logistic regression using only categories available in both the EMR and the BHS. Odds not calculated for Race given significant differences in categories (eg More than one and Not sure not available in EMR).

**Supplemental Table 3. Frequency of Physical Health Chief Complaints Among Youth Presenting to the ED with Past-Week Suicidal Thoughts or Behaviors Detected on Routine Screening, n (%)**

| Chief Complaint | Visits with Past-Week Suicidal Thoughts or Behaviors Detected | | All Visits with a Physical Health Complaint | |  |
| --- | --- | --- | --- | --- | --- |
|  | N | % | N | % | Unadjusted Odds Ratio^a^ |
| All Chief Complaints | 1,179 | 100% | 49,621 | 100% |  |
| Poisoning | 146 | 12.4% | 394 | 0.8% | **46.11 (28.06-75.75)** |
| Abdominal Pain | 144 | 12.2% | 8,273 | 16.7% | 1.39 (0.86-2.25) |
| Injury/Trauma | 142 | 12.0% | 6,015 | 12.1% | **1.89 (1.17-3.07)** |
| STI | 129 | 10.9% | 1,140 | 2.3% | **9.99 (6.13-16.28)** |
| Headache | 78 | 6.6% | 4,635 | 9.3% | 1.34 (0.81-2.22) |
| Respiratory | 73 | 6.2% | 4,066 | 8.2% | 1.43 (0.86-2.38) |
| Chest Pain | 61 | 5.2% | 4,220 | 8.5% | 1.15 (0.68-1.93) |
| GI Symptom | 52 | 4.4% | 3,331 | 6.7% | 1.24 (0.73-2.11) |
| Fainting/Syncope | 38 | 3.2% | 1,880 | 3.8% | 1.62 (0.93-2.81) |
| ENT Symptom | 37 | 3.1% | 2,590 | 5.2% | 1.12 (0.64-1.95) |
| Child Abuse/Assault | 33 | 2.8% | 273 | 0.6% | **10.77 (6.03-19.25)** |
| Urinary/Flank Symptom | 31 | 2.6% | 1,284 | 2.6% | **1.94 (1.09-3.45)** |
| Dermatologic | 27 | 2.3% | 1,912 | 3.9% | 1.12 (0.62-2.03) |
| Diabetes | 23 | 2.0% | 513 | 1.0% | **3.68 (1.99-6.81)** |
| Feeding/Eating Issues | 21 | 1.8% | 951 | 1.9% | 1.77 (0.95-3.31) |
| Fever | 19 | 1.6% | 1,507 | 3.0% | Reference |
| Female GU | 17 | 1.4% | 774 | 1.6% | 1.76 (0.91-3.40) |
| Foreign body | 17 | 1.4% | 178 | 0.4% | **8.27 (4.21-16.22)** |
| Hematologic | 17 | 1.4% | 759 | 1.5% | 1.79 (0.93-3.47) |
| Back/Neck Pain | 15 | 1.3% | 1,170 | 2.4% | 1.02 (0.52-2.01) |
| Seizure | 13 | 1.1% | 597 | 1.2% | 1.74 (0.86-3.55) |
| Altered Mental Status | 12 | 1.0% | 318 | 0.6% | **3.07 (1.48-6.39)** |
| Eye Symptom | 9 | 0.8% | 508 | 1.0% | 1.39 (0.62-3.10) |
| Not categorized | 8 | 0.6% | 542 | 1.1% | 1.07 (0.45-2.56)_ |
| Allergy | 6 | 0.5% | 283 | 0.6% | 1.70 (0.67-4.29) |
| Cardiac | 3 | 0.3% | 283 | 0.6% | 0.84 (0.25-2.85) |
| Male GU | 3 | 0.3% | 302 | 0.6% | 0.79 (0.23-2.67) |
| Non-Specific MSK/Pain | 2 | 0.2% | 365 | 0.7% | 0.43 (0.10-1.86) |
| Fatigue/Weakness | 1 | 0.1% | 125 | 0.3% | 0.63 (0.08-4.75) |
| Limp/Gait concern | 1 | 0.1% | 54 | 0.1% | 1.48 (0.19-11.24) |
| Post-Operative | 1 | 0.1% | 169 | 0.3% | 0.47 (0.06-3.50) |
| Jaundice | 0 | 0.0% | 21 | 0.0% | - |
| Neurological (other) | 0 | 0.0% | 136 | 0.3% | - |

1. Significant associations highlighted in bold.
